# Lockdown or Lockup

**DOI:** 10.1017/dmp.2020.127

**Published:** 2020-12

**Authors:** James J. James

We are currently wrestling with the crucial question of “reopening” America - when and how. The reason for this is that we have seemingly created two competing priorities: saving lives vs restoring the economy. No one can guarantee the best solution to such a complex and rapidly evolving puzzle and we will not be able to truly assess the results of today’s decisions for months or even years to come. With that in mind, what follows is not meant to provide a solution to this conundrum, but to offer some considerations that might assist in helping to formulate a strategy with which to approach it.

Central to the management of this pandemic is determining what Public Health interventions to implement to best contain and mitigate the impacts of COVID-19. Accepting the fact that all interventions have some cost, the goal is to achieve the desired outcome with the least overall harm to society. With COVID-19 the single stated objective has been to “flatten the curve” in order to protect our medical system(s) from being overwhelmed. At the time of implementation, the effects of this strategy on overall mortality and morbidity beyond the potential infectious risks were not calculated.

Because of the extremely high fear levels (fueled by exaggerated worst-case/best-guess models and sensationalized media reporting) in global populations, governments around the world have turned to the use of varying degrees of “lockdowns” as the preferred public health intervention, along with a host of other social distancing requirements. What has been absent is: a) any measure of standardization or even definition of what a lockdown is, b) any clear, quantitative goals, or (c) any objective assessment of the relative costs and benefits of different interventions. For us in the United States, and I presume for other democracies, there are also significant legal and constitutional questions concerning individual liberties and over-arching governmental authorities that have yet to be addressed.

It remains unclear whether there are any added benefits from the extreme lockdown measures (that is, home quarantines and the closing of non-essential businesses and schools) over less restrictive social distancing measures aimed at limiting “risky” personal interactions, especially those involving mass gatherings and large clusters of individuals in enclosed spaces. When the relatively negligible costs of the social distancing measures are compared to the almost catastrophic socio-economic costs of full lockdown the answer to this question becomes imperative. It becomes increasingly difficult to support full lockdowns without supporting evidence of significant benefit beyond that attained from less extreme social distancing interventions. This is especially true given that all epidemic curves eventually flatten out and descend. The current trend of attributing this predetermined outcome to full lockdowns in the absence of supporting evidence sets an extremely dangerous precedent going forward and violates a fundamental tenet of science and logic—correlation is not causation.

Unfortunately, there is no reliable direct evidence other than anecdotal that full lockdowns result in better, equal or worse outcomes than the employment of a variety of social distancing measures. We must therefore turn to the best evidence available at this time, indirect comparisons. One obvious method of interpreting this available information is to compare the epidemiological curves of the 48 countries with 5,000 or more reported cases as of 28 April 2020 (see Worldometers) with the extent of the lockdown, if any, imposed within those nations. There is no single best source for this information but *Business Insider* and Wikipedia are good starting points. This comparison is complicated by the extreme variability of: a) measures imposed and their duration, b) case definitions, c) testing capabilities, d) demographic and geo-political considerations, and e) population compliance. How-ever, even with these confounders, no consistent pattern in terms of the distribution of new cases over time emerges across countries to support full lockdowns, as compared to the implementation of less restrictive measures. Another possible measure of lockdown effectiveness is impact on an epidemiological curve over time. Given that the incubation period for COVID-19 is 1-14 days, with an average of 5 days once a full lockdown is implemented one would expect to see a positive impact on the epidemiologic curve after a two or three week period. This impact is not observed for the hardest hit nations in full lock-down and, as with the indirect comparisons, we find no direct evidence for their use versus less restrictive measures.

Assessing the costs of a full lockdown is also complex and imprecise but it is somewhat more amenable to measurement. In the U.S. we have to date over 20 million newly unemployed and state-wide school closures across the country. Alcohol sales are up; spouse and child abuse reports are increasing; divorces are on the rise; and the backlog of patient needs for mental health services, cancer treatments, dialysis treatments and everyday visits for routine care and even acute emergency services are significantly increasing. All of this will have a significant Public Health impact now and in the future. Add to this the impending global calamity predicted by the UN of losing hundreds of thousands of children to famine alone due to economic collapse of already struggling nations and you precipitate a public health crisis of immeasurable consequence. These are extraordinarily high costs in terms of human lives and socio-economic well-being without a clear demonstration of the benefit derived from the extreme interventions precipitating them.

One of the issues leading to the lockdown strategy was the lack of information as to the true number of individuals infected and the overall ratio of non-susceptible individuals in the population. Recent serology studies out of New York and California along with the results of almost complete viral PCR testing of two COVID-19 exposed populations on the Diamond Princess and Theodore Roosevelt provide evidence that 85% percent of PCR positive individuals. The studies also indicate that up to 85% of an exposed population may be relatively non-susceptible due to the presence of COVID-19 antibodies and/or some as yet undetermined genetic mechanism (for example, some interesting early work seems to indicate that blood type O may be more protective than type A). If accumulating data continue to support a relatively large prevalence of non-susceptibles in a population, then a shift in policy to include herd immunity as a viable mitigation strategy should be revisited. This will both enhance the targeting of public health interventions to the well-defined risk groups, as opposed to the whole population, and greatly diminish concerns about a major resurgence. Another extremely important consideration needs to be addressed, and that is the impact of increased testing on new case counts, which has been a key metric in measuring the progression of the pandemic. Given the relatively high number of sub-clinical to clinical PCR positives, there will be a spike in new cases that are identified as a result of expanded testing and not pandemic progression.

In summary, we are gaining the knowledge and tools to both better protect our medical delivery systems and begin to repair our socio-economic damage. There do not have to be competing priorities, we can address both and maximize lives saved. An important first step is to accept the fact that this is not deciding between lives versus dollars, it is about maximizing a state of physical, mental and social well-being for all.

